# Bias in dyslexia screening in a Dutch multicultural population

**DOI:** 10.1007/s11881-018-0155-0

**Published:** 2018-02-23

**Authors:** Anick Verpalen, Fons Van de Vijver, Ad Backus

**Affiliations:** 1 Department of Social and Behavioral Sciences, Tilburg University, Tilburg, The Netherlands; 2 Bergen op Zoom, The Netherlands

**Keywords:** Culture, Dyslexia screening, Immigrants, Literacy skills, The Netherlands, Word Lexicon

## Abstract

We set out to address the adequacy of dyslexia screening in Dutch and non-western immigrant children, using the Dutch Dyslexia Screening Test (DST-NL) and outcomes of the Dutch dyslexia protocol, both of which are susceptible to cultural bias. Using the protocol as standard, we conducted an ROC (Receiver Operating Characteristics) analysis in Dutch and immigrant third, fifth, and seventh graders, combining a cross-sectional and longitudinal design. Sensitivity and specificity increased with grade, but were non-significant for various subtests in the lowest grade, suggesting considerable non-convergence between the two measures. Effective subtests in all grades, presumably not strongly influenced by Cultural Background or Word Lexicon, were One-Minute Reading, Non-Word Reading, and Nonsense Passage Reading. In a multilevel analysis, cultural background, dyslexia diagnosis, parental education, and grade of first assessment were predictors of subtest performance. In a second analysis, Word Lexicon was added as a proxy of knowledge of the Dutch language and culture. After controlling for Word Lexicon, cultural background became significant for most subtests, suggesting the presence of cultural bias. Subtests assessing technical literacy, such as One-Minute-Reading, Non-Word-Reading, One-Minute-Writing, or Two-Minutes-Spelling, showed more convergence between the two assessments. Less-effective subtests were Naming Pictures, Backward Digit Span, and Verbal and Semantic Fluency. It is concluded that the DST-NL and the standard protocol do not show complete convergence, notably in the lower grades in the multilingual pupil group of our cohort, mainly because dyslexia and literacy difficulties are hard to disentangle.

## Introduction

Many dyslexia screening and assessment tools have been developed to detect literacy difficulties in monolingual children. In Dutch education, the tests are used to identify children with literacy problems, probably caused by dyslexia. Such children then can get further assessment, possibly followed by special treatment for dyslexics, which can enhance school outcomes in various domains, such as academic, vocational, and personal (e.g., self-esteem) (Loykens, Ruijssenaars, Bron, & Van Mameren-Schoehuizen, 2010; Scott, Scherman, & Phillips, 1992). These screening tests are increasingly used in multilingual school populations. Notably in a non-western immigrant context where home and school language are different, extant tests and test procedures can lead to disadvantages and incorrect classifications in these immigrant groups (Everatt et al., 2010).

In the Netherlands, the prevalence of Dutch (majority group) children with dyslexia is about 5%. The same prevalence has been reported for non-western immigrant children (Wentink & Verhoeven, 2004); yet, in the assessment of the latter group, there is a potential problem of misidentification (false positives and false negatives), because it is difficult to recognize dyslexia in children from ethnic groups in which the testing language is not the mother tongue (Cline, 2000; Peer & Reid, 2000). Differences in test scores between Dutch and immigrant children could be a consequence of the groups’ differential knowledge of the Dutch language, unintentional difficulties of an instrument, and family-related factors that impinge on school achievement, such as low socioeconomic status and parental support (O’Bryon, 2014).

The purpose of this study was to identify tasks of the DST-NL which were at risk for bias and we studied the effect of age and schooling on biased tasks. In this study, in which we used a combination of cross-sectional and longitudinal data, we examined the DST-NL subtests and total test score for the plausibility of the presence of bias in the third, fifth, and seventh grade and the effect of schooling and development on DST-NL scores.

## Cultural bias

Fairness in assessment is an issue in diverse groups of children. Unintentional cultural factors can influence the way children interpret test items and respond to them. These factors with an adverse impact on test scores of usually immigrant children are referred to as bias (Solano-Flores, 2011; Van de Vijver & Leung, 1997). The presence of cultural specificity in a test or test item could introduce bias. The stronger the cultural specificity of a test or test item, the larger the likelihood of the items are biased against those outside the majority culture. For example, in a test of crystallized knowledge, the test item “Who was the first president of the United States?” could represent a biased item that favors European-American children and could be more difficult for an immigrant child from Paraguay (Reynolds & Brown, 1984).

## Types of bias

In cross-cultural psychology, three types of bias have been distinguished: construct bias, method bias, and item bias (Van de Vijver & Leung, 1997; Van de Vijver & Poortinga, 2005). Construct bias occurs if the construct measured is not identical across cultural groups or if there is an incomplete overlap of indicators associated with the construct across cultural groups (e.g., poor sampling of all relevant behaviors or differential appropriateness of the behaviors associated with the construct or incomplete coverage of the construct). Another type of bias is called method bias. Method bias refers to measurement anomalies that are related to the administration of an instrument (differential familiarity with stimulus materials or response procedures, differences in environmental administration conditions, incomparability of samples caused by differences in education, or other background characteristics) (Van de Vijver & Leung, 1997; Van de Vijver & Poortinga, 2005). The third type of bias, item bias or Differential Item Functioning, refers to anomalies at item level, caused by poor item translation or inadequacy of item content in a cultural group (Van de Vijver & Leung, 1997; Van de Vijver & Poortinga, 2005). An item about bacon was more difficult for Islamic children than for Dutch children, because they have less or no contact with it (Van de Vijver & Leung, 1997). Culture, language, age, education, socioeconomic status, and acculturation can all play an important role in test performance (O’Bryon, 2014), especially when tests measure a language-related construct, such as learning disability or dyslexia.

## Language

Many tests and assessments depend on language in their administration (instructions, item contents, and response procedures). Language proficiency is often not the target of assessment although it may influence the test results (Trumbull & Solano-Flores, 2011). A limited proficiency in the majority language makes the assessment procedure more difficult for immigrant children. Low performance of immigrant children may be due to a lack of understanding of the language of the test rather than a lack of content knowledge. Immigrant children have a dual challenge, because they have to develop their majority language skills and they have to learn the academic content of the curriculum in the majority language (Abedi, 2011; Hakuta, Butler, & Witt, 2000).

Differences between monolinguals (majority group members) and immigrant children tend to be larger on verbal fluency tests than on letter fluency tests (Gollan, Montoya, & Werner, 2002; Verpalen & Van de Vijver, 2011); moreover, immigrant children have a smaller vocabulary size in the mainstream language (Hamers & Blanc, 2000), recognize fewer difficult vocabulary words, and have more tip-of-the-tongue (just-cannot-remember-the-word) retrieval failures than monolinguals (Gollan & Brown, 2006). They also name pictures slower than monolinguals and name fewer pictures correctly on standardized naming tests (Gollan & Brown, 2006; Roberts, Garcia, Desrochers, & Hernandez, 2002; Verpalen & Van de Vijver, 2011). These disadvantages are also present when bilinguals are tested in their first language (Ivanova & Costa, 2008). Developing a majority language and particularly the academic register of the majority language as a second language takes much effort from 5 to 7 years and usually even after that period (Abedi, 2011; Hakuta et al., 2000).

## Sociocognitive factors

In Europe and the USA, migration is a major factor in bilingualism (O’Bryon, 2014; Tabouret-Keller, 2006). Poor linguistic and scholastic results of immigrants tend to be related with a low level of proficiency in the majority language and less favorable sociocultural factors (Backus, 2013; Hamers & Blanc, 2000; O’Bryon, 2014). In the Netherlands, 21% of the population has at least one foreign-born parent (9% in a Western country and 12% in a non-Western country) (Statistics Netherlands, 2014). The educational achievements of non-Western immigrant children are below those of Dutch mainstream children; fewer students enter forms of higher education but an increase in the percentage of non-Western immigrant children entering higher levels of secondary and tertiary education is reported in the Netherlands for the last 10 years (Backus, 2013; Statistics Netherlands, 2007, 2012). Compared to their mainstream peers, non-Western immigrant children are more likely to grow up in families with low levels of education, to live in an unstable neighborhood and in relative poverty which could harm their academic achievement (O’Bryon, 2014; Statistics Netherlands, 2014). Researchers showed in studies with Arabic immigrant children in the USA and Russian-Jewish immigrant children in Israel that the level of parental education before immigration and their socioeconomic status after immigration play a larger role in the child’s successful second language development than the parents’ second language proficiency (Kenny, 1996; Schwartz, 2012; Schwartz, Kozminsky, & Leikin, 2009). In the Netherlands, a small association has been found between parental education in the home country and immigrant children’s reading ability (Onderwijsraad, 2013).

## Reading and literacy development in bilingual children

Progress in acquiring literacy in the majority language by immigrant children depends on social, political, and educational factors, the child’s exposure to the majority language and academic culture, and on literacy skills and experience developed in their first language, if their mother tongue has a script. Success of early literacy acquisition is related to the value attached to literacy at home and the level of literacy support in the home environment (Cobo-Lewis, Pearson, Eilers, & Umbel, 2002; Francis, 2012; Verhoeven & Durgunoğlu, 1998).

Immigrant children, whose mother tongue is a minority language, need to learn literacy skills in the majority, second language, which they often do not speak well, notably in the early schooling years (Bialystok, 2001). In this situation, the cognitive skills associated with literacy are being learned at the same time as the linguistic system that is encoded in writing. Reading development has some universal components across languages. When children learn to read, they have to recognize which language elements are encoded in the writing system (the general mapping principle) and deduce exactly how these elements are encoded (the mapping details). These skills and knowledge do not involve language-specific aspects, such as specific language elements or sounds, but universal principles, such as mapping a sound to a symbol.

When these skills are developed in one language, they are available and functional for development in another language (Koda, 2008). Knowing how to read in the first language can facilitate literacy development in the second language (Bialystok, 2001) as children can transfer their skills and knowledge to literacy in a new language (Cisero & Royer, 1995; Durgunoğlu, Nagy, & Hancin-Bhatt, 1993; Gottardo, Yan, Siegel, & Wade-Woolley, 2001; Verhoeven, 1994). Children’s progress in literacy can be adversely influenced by a lack of age-appropriate competence and academic skills in either their first language or second language, which impedes the development of the cognitive systems needed to function academically (Cisero & Royer, 1995; Durgunoğlu et al., 1993; Gottardo et al., 2001; Verhoeven, 1994). Literacy skills can only progress with second language proficiency, which is influenced by training in the language (contact hours in the second language). Learning a second language when the script is different from the first language or when the mother tongue has no script at all can affect the progress at least initially if literacy development in the second language starts later (Barlett, 2001). As a consequence, dyslexia screening tests could be least useful when the role of assessment is most important: for early identification to start specific treatment and early intervention. On the other hand, recent researchers (e.g., Durgunoğlu et al., 1993; Geva, 2000) have shown also a growing evidence of a weak relationship between the word-based reading process in the second language (the technical skill, and not the comprehension skill) and the oral second language proficiency. Durgunoğlu et al. (1993) demonstrated that, unlike oral language, phonological skills predicted technical reading skills (word recognition and pseudo-word reading). Other researcher studied children with different writing systems as first language and showed also weak relationships between the proficiency in the second language and word recognition and pseudo-word reading (Geva & Clifton, 1993; Geva & Siegel, 2000; Gholamain & Geva, 1999). In line with these findings, Geva (2000) reported that when children have been exposed to literacy instruction, they are able to decode words, even when their second language proficiency is still developing.

## Assessing literacy skills in immigrant children

The educational home environment, language, and literacy ability, poverty, and parental education of mainstream and immigrant children are often different. A specific group of immigrants, such as refugee children, could have no, limited, or disrupted schooling and experience with food scarcity, displacement, and traumas. These factors can contribute to immigrants’ underachievement in reading and spelling (Ehntholt, Smith, & Yule, 2005; Limbos & Geva, 2001; Pollard-Durodola, Cárdenas-Hagan, & Tong, 2014). Underachievement makes it difficult to distinguish between immigrant children who are developing normally with basic weaknesses in their language abilities and immigrant children who are experiencing reading failure (Geva, 2000). This ambiguity can lead to a “wait-and-see” approach in schools because standardized assessment measures typically do not indicate to what extent low test scores of immigrants are an indication of low reading achievement or language and learning difficulties due to their different background (Chiappe, Siegel, & Gottardo, 2002; Gersten & Baker, 2003). Researchers have found that oral language proficiency only plays a marginal role in reading skills (e.g., Durgunoğlu et al., 1993; Geva, 2000; Geva & Clifton, 1993; Geva & Siegel, 2000; Gholamain & Geva, 1999). Next to these findings, the development of phonological awareness skills and related processes like naming speed and auditory memory, orthographic knowledge, and speed of lexical access are strongly related to individual differences in word reading skills, which presumably are universal cognitive and linguistic factors that can predict reading ability in both the first and second language (Durgunoğlu et al., 1993; Geva, 2000; Geva & Siegel, 2000). Phonological awareness is the ability to reflect upon and manipulate phonological units in a language (Kuo & Anderson, 2008). Children with dyslexia have reading and spelling problems but they also experience difficulties with phonological tasks, phonological short-term memory tasks, and rapid automatized naming tasks (Blomert, 2006; Goswami, 2008). Two of the best indicators of early reading problems and dyslexia in both the first and second language are deficits in phonological awareness and rapid naming (Limbos & Geva, 2001; Paulesu et al., 2001). These indicators are assessed in most of the dyslexia screening tests, also in the Dutch Dyslexia Screening Test.

## Present study: bias in the Dyslexia Screening Test NL

Dyslexia assessment is possible in young children; several dyslexia indicators become manifest before the child learns to read. Researchers have found that young preschool children who later developed dyslexia showed difficulties in pre-literacy skills such as phonological awareness and letter knowledge in preschool which predict later reading ability (Elbro & Petersen, 2004; Regtvoort & Van der Leij, 2007; Van Otterloo, Van der Leij, & Henrichs, 2009). The Dyslexia Screening Test (Dutch version: DST-NL) is a well-known instrument for identifying children at risk for dyslexia (Kort et al., 2005). The DST-NL is a screening test; an “at risk score”, derived from an administration of the test, indicates that the presence of dyslexia might be the underlying problem of literacy difficulties. Full assessment (with additional tests) of such “at risk” children is necessary to diagnose dyslexia (Kort et al., 2005). The DST-NL is a Dutch instrument, translated from English, with a target age range from 6.5 to 16.5 years (Fawcett & Nicolson, 2005). The English edition was published in 1996 and revised in 2004. In the 1996 version, the phonological tasks were not part of the “at risk score” (named PLQ in this version). The revised edition is divided in two versions: the DST-Junior for primary school-aged children (6.6 to 11.5 years) and DST-Secondary for secondary school-aged children (11.6 to 16.5 years). Two subtests are added to each version (Rhyme and Vocabulary for primary school, Spoonerisms and Non-Verbal Reasoning for secondary school). In the new edition, the phonological tasks are part of the “at risk score” (called ARQ in the new edition), in contrast to the older edition; this change is in line with the important role of phonological awareness deficits in detecting dyslexia (Blomert, 2006; Goswami, 2008; NRD, 2013). The Dutch edition is a translation of the first edition of the English DST, normed in a Dutch population. There is no translation of the new edition available in the Netherlands. We followed the 1996/2004 recommendation not to include the tests in the PLQ score, although, as shown below, we also addressed their suitability for assessment in multicultural populations. The DST-NL assesses skills that play an important role in dyslexia: literacy skills, rapid naming, working memory, phonological awareness, reading ability, and spelling ability. Many verbal subtests of the DST-NL have references to the Dutch culture (e.g., Dutch names) and could be more difficult for immigrant children, even the rapid naming and verbal fluency tests as described in the introduction.

For our study, we also had the outcome of the Dutch dyslexia protocol (NRD, 2013) available. In this protocol, the dyslexia criteria are described and instruments for assessment are advised in its addition. Following this protocol, the child must have serious reading problems (percentile score < 10) or serious reading difficulties (percentile score < 16), combined with serious spelling problems (percentile score < 10) and two additional very low scores (percentile score < 10) on the dyslexia indication accuracy and speed of phonological processing, accuracy and speed of sound-letter mapping, and speed of naming digits and/or numbers to diagnose a child as dyslexic (NRD, 2013). We cannot rule out that the dyslexia protocol is susceptible to the same cultural bias as the DST-NL. As a consequence, there is no golden standard against which to evaluate these measures. For the purpose of the analysis, we used the dyslexia protocol scores as validity standard, because using the protocol as (fallible) standard against which the DST can be compared allows the use of various tools such as sensitivity and specificity analysis, which allow for a study of the convergence of the DST-NL and dyslexia protocol outcomes.

In this study, we tried to examine to what extent it is possible to detect Dutch and immigrant children at risk for dyslexia with the same instrument (DST-NL) and tried to make a reasonable case for the decrease of cultural bias in the DST-NL for immigrant children across the third, fifth, and seventh grade, taking into account the differences in stage of reading development per grade. The stages are derived from the triangle framework of normal reading development and visual word recognition (Seidenberg & McClelland, 1989), complemented by more recent findings from Bishop and Snowling (2004), Glenberg, Goldberg, and Zhu (2009), Marly, Szabo, Levin, and Glenberg (2011) and Welsby and Pexman (2014) (see Verpalen & Van de Vijver, 2015, for a more elaborate explanation of this framework). These stages are useful for monolingual and bilingual children if they started education at least in the first and second grade in the Netherlands (kindergarten). In the third grade, the child is a starting reader using letter-sound mapping (via the phonological pathway); in the fifth grade, the child uses more word recognizing skills with direct activation of the meaning of the word via the semantic pathway. This grade often coincides with a switch in language dominance from the mother tongue to the majority second language, which is generally claimed after the age of 8 to characterize immigrant children who were exposed to the second language at the age of about 2 (e.g., when they started kindergarten) (Akinci, Jisa, & Kern, 2001). In the seventh grade, the children tend to be fluent readers (using the semantic pathway).

We used a combined cross-sectional and longitudinal design in which a subsample is assessed twice (third and fifth or fifth and seventh grade) or thrice (third, fifth, and seventh grade). The development in vocabulary knowledge and cultural knowledge could have a positive effect on DST-NL scores throughout the school years. Dyslexia was independently assessed by psychologists using a comprehensive test battery according to the official Dutch protocol (Blomert, 2006; NRD, 2013). To clarify the role of cultural bias in dyslexia screening tasks, the following hypotheses are tested: first, the prediction of dyslexia diagnosis using the DST-NL subtests and therefore the DST-NL risk score is less accurate for immigrant children in the third, fifth, and seventh grade than for Dutch children in the same grades (due to method and item bias). Immigrant children’s underachievement can make it more difficult to interpret the test scores, with more false positives as a consequence. Second, the convergence between the accuracy of the prediction of the DST-NL subtests scores and therefore the DST-NL risk score and the dyslexia protocol outcomes increase throughout the years of schooling for the immigrant children. Third, the verbal subtests of the DST-NL are more difficult for immigrant children in the third, fifth, and seventh grade, even after controlling for the level of Word Lexicon, Parental Education, and the grade of first assessment (cultural bias).

## Method

### Participants

Dutch children start their education in the first and second grade of primary school (kindergarten) where some occasional teaching takes place. Teaching at a larger scale starts in the third grade when children are 6 years of age. This study is part of a larger project and is the last study of three studies. In the first study, bias in the DST-NL was examined in Dutch and immigrant children of the fifth grade (Verpalen & Van de Vijver, 2011), while in the second study, bias was examined in the DST-NL comparing Dutch and immigrant fifth and seventh graders (Verpalen & Van de Vijver, 2015). Data from the first and second study and the newly collected data were used as one dataset in the current study. In 2006, data collection started in the fifth grade for the first study. Between 2008 and 2013, children of the third, fifth, and seventh grade were assessed for the second and current study. In these 7 years, the cohort was enlarged and changed. As a consequence, shifts occurred in the school populations from which we recruited. Some children of the first and second study, who were originally in the non-dyslexic group, were diagnosed as dyslexic and added to the dyslexic group (and deleted from the non-dyslexic group) of the current study; some children were diagnosed with low intelligence or weak memory function and deleted from the cohort in the fifth or seventh grade; some children moved to another country or district; finally, some children moved into the district of the schools of this cohort and were added to the third, fifth, or seventh grade. The main reasons for attrition were relocation within the Netherlands and remigration. In one of the participating schools, the sample size increased because of a merger with another school, where the DST-NL was not administered before.

The DST-NL was administered to 324 (145 Dutch and 179 non-western immigrant) children in a period of 7 years (169 boys and 155 girls). Data were available of 128 children of the third grade (43 Dutch and 85 immigrant children), 244 children of the fifth grade (97 Dutch and 147 immigrant children), and 201 children of the seventh grade (98 Dutch and 103 immigrant children) (Table 1).

**Table 1:** Differences in standardized mean scores reading and spelling test per grade and school between location A and location B

	3rd grade						5th grade						7th grade					
	Location A (*n =* 84)		Location B (*n =* 99)				Location A (*n =* 84)		Location B (*n =* 99)				Location A (*n =* 84)		Location B (*n =* 99)			
	*M*	SD	*M*	SD	*F*	*ƞ* ^*2*^	*M*	SD	*M*	SD	*F*	*ƞ* ^*2*^	*M*	SD	*M*	SD	*F*	*ƞ* ^*2*^
Reading school Test	3.79	1.25	3.90	1.18	.40	.00	3.65	1.24	3.66	1.28	.00	.00	4.04	1.29	3.91	1.33	.42	.00
Spelling school Test	3.63	1.13	4.10	1.03	8.71	.05^**^	2.98	1.27	3.35	1.35	3.75	.02*	3.60	1.21	3.68	1.07	.23	.00

*p < .05, **p < .01

In the Dutch third grade, the children were aged 6–7 years, in the fifth grade, 8–9 years, and in the seventh grade, 10–11 years. Almost all (95%) of the immigrant children were second or third generation, 44% of the immigrant children were Turkish, 33% were Moroccan, and 23% had other countries of origin (such as Iraq, Vietnam, Indonesia, Brazil, various countries in Eastern-Europe, and in Africa). A small number of them were refugees. Almost all immigrant children had started education at the age of two (preschool) or four (kindergarten). Twenty-one percent of the Dutch and 11% of the immigrant participants were diagnosed with dyslexia in reading and spelling by psychologists from different centers outside the school. The used test battery measures dyslexia indications (reading ability, spelling ability, phonological awareness, and rapid naming), according to the official Dutch dyslexia protocol (Blomert, 2006) and the accompanying cut-off criteria (NRD, 2013). Although researchers have shown that some dyslexics had also verbal short-term and working memory difficulties (Durgunoğlu et al., 1993; Geva, 2000; Geva & Siegel, 2000; Nicolson & Fawcett, 2008), the cut-off criteria of this protocol were only based on technical reading and spelling skills, phonological skills, and rapid naming skills. The number of dyslexic children in the sample is relatively high, because the school specializes in dyslexia care in the curriculum. The school pays the full assessment of all children at risk for dyslexia, which is exceptional in Dutch education. Pupils were attending a public school; in the Netherlands, 0.6% of primary and secondary schools are private schools and 99.4% are public schools. Parents decide on the school to which to send their children and often choose the school where we conducted our study because of the opportunity of specialized dyslexia treatment inside the school.

## Measures

The Dutch version of the DST-NL was administered in a quiet room by two remedial teachers who were not their teacher and a school psychologist. The test has 14 subtests (standardized scores from 1 to 19); the risk indicator (called PLQ, Psycho Linguistic Quotient) is based on only seven subtests: Rapid Naming Pictures, Rapid Naming Letters, One-Minute Reading, Two-Minutes Spelling, Nonsense Passage Reading, Non-Word Reading, and One-Minute Writing. The other subtests are an indication of memory functioning and phonological awareness (Phonemic Segmentation 1 and 2, and Backward Digit Span) and Association (Verbal Fluency and Semantic Fluency). Although these subtests are not part of the risk indicator of the DST-NL, phonological awareness still provides a good indicator of dyslexia (Blomert, 2006; NRD, 2013). The subtests, Postural Stability and Bead Threading (Physical Ability), were not administered because Kort et al. (2005) reported a non-significant relationship in their Dutch norm group between Physical Ability and dyslexia (*r* = − .11, *ns*). The correlations (absolute value) between Postural Stability and Bead Threading and the other subtests were all < .20 in the Dutch norm group (Kort et al., 2005). Finally, the subtest, Postural Stability, could also be experienced as unpleasant because children are blindfolded and get a push in the back.

There were differences in the language usage at home in the immigrant group; all children were asked which language they speak at home and how often; 27% of the immigrant children used only the mother tongue at home (scored as level 1), 33% used more mother tongue than the Dutch language (level 2), 17% used half mother tongue and half Dutch (level 3), 18% spoke more Dutch than the mother tongue at home (level 4), and 4% of the immigrant children used only the Dutch language at home (level 5). The predominance of Dutch spoken at home correlated significantly with the level of Word Lexicon school test score: *r* = .30, *p* < .001. Both parents of the Dutch monolingual children had Dutch as their first language.

Most immigrant participants did not have good Dutch vocabulary knowledge. The level of Dutch vocabulary knowledge (assessed with the same school vocabulary tests at both schools) was divided in five level groups, ranging from very low (score 1) to high (score 5) (see Table 3 for the standardized mean scores). Word Lexicon scores were significantly higher for the majority group with a large effect (effect size *r*: small effect size *r* = .15; medium effect *r* = 30; large effect size *r* = .50; Cohen, 1988) in the third (Dutch *M* = 3.97, SD = 1.00, immigrant *M =* 2.08, SD *=* 1.19, *t*(299) = 14.97, *p* < .001, *d* = 1.73, *r* = .65), fifth (Dutch *M* = 3.74, SD = 1.09, immigrant *M =* 2.12, SD *=* 1.15, *t*(285) = 12.134, *p* < .001, *d* = 1.44, *r* = .58), and seventh grade (Dutch *M* = 3.67, SD = 1.06, immigrant *M =* 2.43, SD *=* 1.05, *t*(211) = 8.55, *p* < .001, *d* = 1.18, *r* = .51). In an ANOVA with Culture (Dutch vs. immigrant) and grade as fixed factors and Word Lexicon as dependent variable, the effect of Grade was not significant (as expected because of the standardized scores across grades, mentioned above); the effect of Culture was significant and large (*F*(5, 315) = 98.03, *p* < .001, *η*^2^ = .24). The interaction between Grade and Culture was significant, yet small (*F*(5, 315) = 3.79, *p* < .05, *ƞ*^2^ = .02), which referred in this case to a decrease per grade in the Dutch group and an increase per grade in the immigrant group (Table 3), which makes the differences in mean scores smaller over the years.

The level of parental education (i.e., the educational level in the home country of the parents) is divided in three groups: low (score 1: no education or only primary school), medium (score 2: primary school and 3 years of low level of high school), and high (score 3: at least 4 years of middle or high school). In this study, 2% of the Dutch and 53% of the immigrant parents had a low educational level in their home country, 14% of the Dutch and 15% of the immigrant parents had a medium educational level in their home country, and 83% of the Dutch and 32% of the immigrant parents had a high educational level in their home country. The differences in mean scores of the level of parental education in the Dutch and immigrant group was significant, with Dutch parents having a higher level of education, *χ*^2^(2, *N* = 324) = 106.68, *p <* .001.

A combined longitudinal and cross-sectional design was used. Some children were assessed in one grade, some in two grades, and others in three grades, depending on their presence enrolment in school. This combined design enabled the use of all data available (thereby enlarging sample size and power in our statistical tests) and to model individual growth (rather than confounding growth and cohort differences). The period between test and retest was 2 years or more; therefore, we expected the effect of memory at the retest not to be very strong (Neyens & Aldenkamp, 1999).

## Results

No differences were found in mean scores between boys and girls in our population, with one exception: boys scored significantly lower on One-Minute Writing (9.71) than girls (10.32); this finding is difficult to interpret as an ANOVA design with the presumably relevant control variables (dyslexia diagnosis and cultural group) has a small sample size at cell level, which precludes an adequate analysis of the gender difference. The Word Lexicon school test (Cito LOVS Word Lexicon), Reading school test (Cito LOVS DMT), and the spelling school test (Cito LOVS Spelling) were administered in January in each grade. The levels of Word Lexicon, Spelling, and Reading were divided in line with the test norms in five classification groups, based on the standardized scores across grades, ranging from very low (score 1) to very high (score 5). An overview of mean scores on DST-NL and school tests of the Dutch and immigrant, non-dyslexic and dyslexic children in the third, fifth, and seventh grade is shown in Tables 2 and 3.

**Table 2:** Standardized mean scores (scaled 1–19) and standard deviations of Dutch and immigrant non-dyslexic and dyslexic third, fifth, and seventh graders

DST subtest	3ʳͩ graders				5ͭͪ ͪ graders				7ͭͪͪͪͪ ͪͪͪͪ graders			
	Dutch		Immigrant		Dutch		Immigrant		Dutch		Immigrant	
	Non-dyslexic (*n =* 34) *M* (SD*)*	Dyslexic (*n =* 9) *M* (SD)	Non-dyslexic (*n =* 75) *M* (SD)	Dyslexic (*n =* 10) *M* (SD)	Non-dyslexic (*n =* 76) *M* (SD)	Dyslexic (*n =* 21) *M* (SD)	Non-dyslexic (*n =* 128) *M* (SD)	Dyslexic (*n =* 19) *M* (SD*)*	Non-dyslexic (*n =* 74) *M* (SD)	Dyslexic (*n =* 24) *M* (SD)	Non-dyslexic (*n =* 89) *M* (SD)	Dyslexic (*n =* 14) *M* (SD)
Naming Pictures	10.24	9.33	9.44	8.30	9.99	8.95	9.68	8.05	9.39	8.92	9.63	7.79
	*(3.17)*	*(2.45)*	*(3.39)*	*(2.54)*	*(2.67)*	*(3.22)*	*(3.12)*	*(2.22)*	*(2.41)*	*(2.50)*	*(2.66)*	*(2.33)*
Naming Letters	11.56	9.22	11.09	9.40	11.62	9.24	9.62	7.84	11.77	10.00	10.67	7.21
	*(2.60)*	*(3.77)*	*(2.98)*	*(2.01)*	*(2.78)*	*(3.36)*	*(2.97)*	*(2.04)*	*(2.74)*	*(2.75)*	*(3.04)*	*(2.29)*
One-Minute Reading	9.06	4.67	8.27	4.70	10.71	5.67	9.69	5.84	11.35	6.67	10.71	6.29
	*(4.24)*	*(1.32)*	*(3.27)*	*(1.16)*	*(2.72)*	*(2.20)*	*(2.66)*	*(1.89)*	*(2.33)*	*(2.14)*	*(2.37)*	*(2.23)*
Phonological Segmentation 1	9.97	8.78	9.36	8.50	10.26	7.95	9.51	8.05	9.65	6.71	8.62	7.14
	*(2.56)*	*(1.79)*	*(2.70)*	*(2.76)*	*(2.44)*	*(2.52)*	*(2.28)*	*(2.48)*	*(2.02)*	*(2.29)*	*(1.81)*	*(2.18)*
Phonological Segmentation 2	9.85	8.78	9.11	9.00	10.45	7.48	9.30	7.11	9.36	7.58	8.67	6.86
	*(1.86)*	*(.67)*	*(1.56)*	*(.00)*	*(2.08)*	*(2.18)*	*(2.02)*	*(2.42)*	*(1.98)*	*(1.77)*	*(1.72)*	*(1.92)*
Two-Minutes Spelling	10.44	8.11	9.71	8.80	10.16	6.81	9.88	7.95	10.07	7.67	9.92	7.50
	*(1.83)*	*(2.62)*	*(2.16)*	*(1.32)*	*(2.20)*	*(2.46)*	*(2.23)*	*(2.27)*	*(2.39)*	*(1.69)*	*(1.90)*	*(2.14)*
Backward Digit Span	9.50	9.11	8.87	8.20	10.34	9.10	10.20	10.95	10.58	10.00	10.13	10.29
	*(2.35)*	*(3.02)*	*(2.74)*	*(3.62)*	*(2.93)*	*(2.76)*	*(2.67)*	*(2.61)*	*(2.36)*	*(2.59)*	*(2.62)*	*(2.64)*
Nonsense Passage Reading	10.03	7.89	9.85	8.40	10.62	7.14	10.73	7.21	9.55	6.00	10.42	6.79
	*(2.26)*	*(1.97)*	*(2.06)*	*(1.08)*	*(2.26)*	*(3.05)*	*(2.10)*	*(1.99)*	*(2.37)*	*(1.69)*	*(2.31)*	*(3.07)*
Non-Word Reading	9.44	5.67	9.00	6.60	9.37	4.67	10.02	4.95	10.50	5.50	10.92	6.21
	*(2.92)*	*(1.58)*	*(2.54)*	*(1.58)*	*(2.26)*	*(2.39)*	*(1.90)*	*(2.17)*	*(2.31)*	*(2.32)*	*(2.02)*	*(1.42)*
One-Minute Writing	11.00	9.00	10.37	8.80	10.14	7.05	10.05	8.00	10.55	7.87	10.33	9.07
	*(2.70)*	*(2.00)*	*(2.54)*	*(3.23)*	*(2.65)*	*(2.82)*	*(2.87)*	*(3.02)*	*(2.49)*	*(2.59)*	*(2.63)*	*(2.84)*
Verbal Fluency	10.94	7.89	10.37	11.30	11.26	8.90	10.25	11.37	11.12	9.96	10.87	10.71
	*(2.87)*	*(3.52)*	*(3.53)*	*(3.68)*	*(3.04)*	*(3.33)*	*(2.79)*	*(2.79)*	*(2.29)*	*(3.30)*	*(2.37)*	*(1.54)*
Semantic Fluency	10.74	10.33	9.03	10.00	10.82	9.57	9.16	9.26	10.27	9.50	8.64	9.29
	*(2.59)*	*(2.65)*	*(2.84)*	*(2.40)*	*(2.38)*	*(1.99)*	*(2.44)*	*(1.97)*	*(2.27)*	*(1.62)*	*(2.43)*	*(2.49)*
PLQ risk score	102.44	83.89	98.01	85.20	102.62	78.86	99.58	79.58	102.47	82.62	102.49	81.14
	*(15.89)*	*(10.66)*	*(14.61)*	*(7.80)*	*(12.78)*	*(14.44)*	*(12.98)*	*(10.88)*	*(11.83)*	*(10.36)*	*(10.39)*	*(9.38)*

**Table 3:** Standardized mean scores (scaled 1–19) and standard deviations of Dutch and immigrant non-dyslexic and dyslexic third, fifth, and seventh graders (all assessments of all participated children during their education career)

School test	3ʳͩ graders				5ͭͪ ͪ ͪ graders				7ͭͪͪͪͪ ͪͪͪͪ graders			
	Dutch		Immigrant		Dutch		Immigrant		Dutch		Immigrant	
	Non-dyslexic (*n* = 103) *M* (SD)	Dyslexic (*n =* 26) *M* (SD)	Non-dyslexic (*n =* 148) *M* (SD)	Dyslexic (*n =* 19) *M* (SD)	Non-dyslexic (*n =* 90) *M* (SD)	Dyslexic (*n =* 26) *M* (SD)	Non-dyslexic (*n =* 141) *M* (SD)	Dyslexic (*n =* 19) *M* (SD)	Non-dyslexic (*n =* 76) *M* (SD)	Dyslexic (*n =* 24) *M* (SD)	Non-dyslexic (*n =* 100) *M* (SD)	Dyslexic (*n =* 14) *M* (SD)
Reading school test	3.96 *(1.15)*	2.69 *(1.12)*	3.79 *(1.20)*	2.21 *(.98)*	3.96 *(1.06)*	1.68 *(.85)*	3.84 *(1.01)*	1.74 *(.81)*	4.36 *(.92)*	1.96 *(1.23)*	4.29 *(.97)*	2.14 *(1.17)*
Spelling school test	4.13 *(.98)*	3.12 *(1.34)*	3.72 *(1.11)*	3.53 *(1.26)*	3.48 *(1.29)*	1.88 *(1.07)*	3.43 *(1.15)*	1.68 *(.75)*	4.01 *(.89)*	2.75 *(1.07)*	3.69 *(1.04)*	2.29 *(.83)*
Word lexicon school test	3.97 *(1.05)*	3.96 *(.82)*	2.03 *(1.20)*	2.47 *(1.07)*	3.85 *(1.02)*	3.31 *(1.26)*	2.10 *(1.16)*	2.26 *(1.10)*	3.72 *(1.12)*	3.50 *(.83)*	2.37 *(1.04)*	2.86 *(1.03)*

## Hypothesis testing

To evaluate the association of the DST-NL with the dyslexia diagnosis and test the three hypotheses, an ROC (Receiver Operating Characteristics) analysis was calculated for each subtest and the PLQ, for the Dutch and immigrant third, fifth, and seventh graders, using the standardized scores (mean scores see Tables 2 and 3). To enlarge the number of participants in the subgroups (non-dyslexic or dyslexic, Dutch or immigrant, and third, fifth or seventh graders), the repeated measures are included. The ROC curve plots test sensitivity on the vertical axis against its false positive rate (1-specificity rate) on the horizontal axis. The basic measures of performance of diagnostic tests are constituted by sensitivity (the true positive rate) and specificity (the true negative rate). To interpret the ROC curves, a combined measure of sensitivity and specificity is calculated: the area under the ROC curve (AUC). The AUC, with a value between 0 and 1, is interpreted as the average value of sensitivity for all possible values of specificity. The closer the AUC is to 1, the better the overall diagnostic performance of the test; a test with an AUC value of 1 is perfectly accurate. An AUC value is acceptable if .70 ≤ AUC < .80, excellent if .80 ≤ AUC < .90, and outstanding if AUC ≥ .90 (Lammers, Pelzer, Hendrickx, & Eisinga, 2007). An asymptotic significance below .05 is interpreted as showing that the discrimination power of the subtest is better than guessing.

**Fig. 1:**
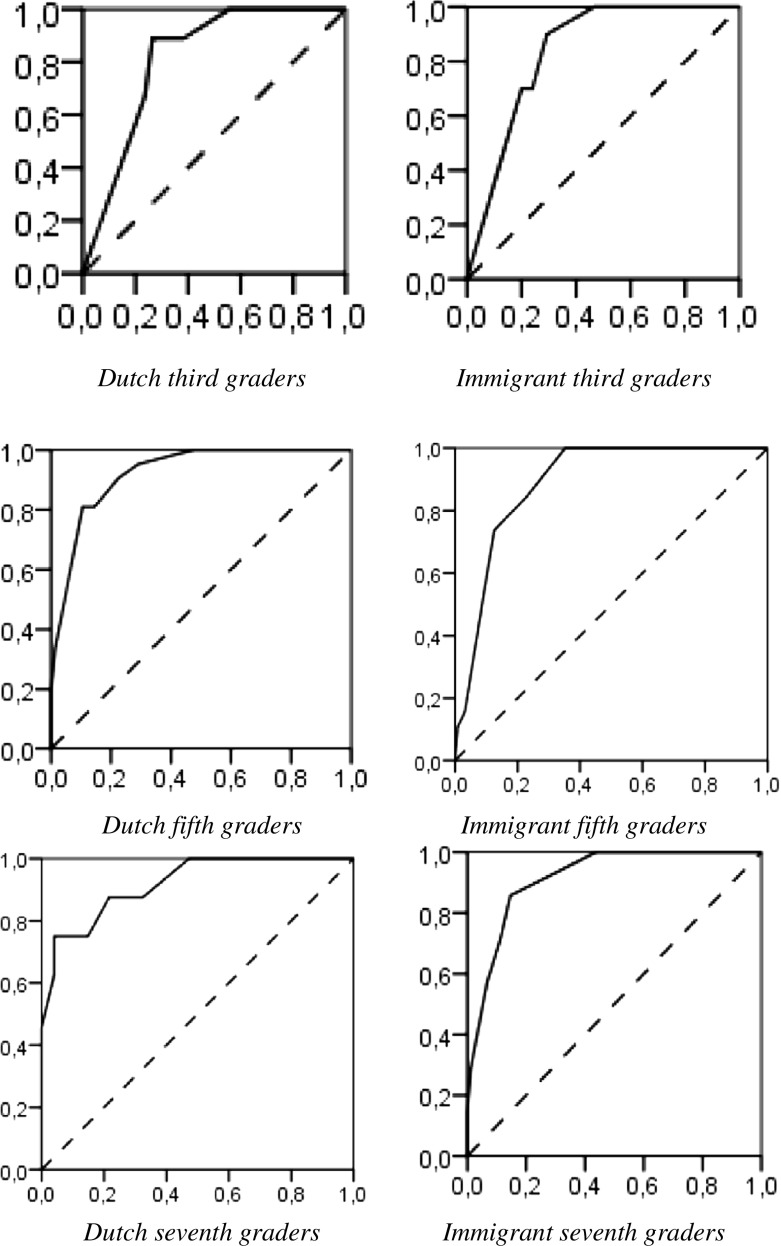
ROC curves DST subtest Word Reading for Dutch and immigrant third, fifth, and seventh graders

In this research, different AUC values were found for the DST-NL subtests and PLQ scores in the groups, which were compared (all Dutch and all immigrant children who were assessed in respectively the third, fifth, and seventh grade) (Table 4). Three subtests, Word Reading, Nonsense Passage Reading, and Non-Word Reading, had an acceptable and significant AUC value above .70 for Dutch and immigrant children in all three grades (third, fifth, and seventh). This means that only these three DST-NL subtests combine sensitivity and specificity in an adequate manner for all children in each grade (and show considerable convergence with the dyslexia protocol outcomes), which confirms hypothesis 1. The PLQ had also a significant AUC value above .70 in all three grades. The AUC value of Word Reading is excellent in the third grade (Dutch children: AUC = .81, immigrant children: AUC = .84, see Fig. 1 and Table 4), in the fifth grade (Dutch children: AUC = .92, immigrant children: AUC = .89, see Fig. 1 and Table 4), and in the seventh grade (Dutch children: AUC = .92, immigrant children: AUC = .92, see Fig. 1 and Table 4 for all the AUC values of the DST-NL subtests and the PLQ). This finding shows that the PLQ and protocol outcomes showed important convergence.

**Table 4:** AUC (area under the curve) scores and their significance per subtest and PLQ risk score, all assessments (including first-, two-, and three times tested) with the DST

	3th grade				5th grade				7th grade			
	Dutch		Immigrant		Dutch		Immigrant		Dutch		Immigrant	
	Area	*p*	Area	*p*	Area	*p*	Area	*p*	Area	*p*	Area	*p*
*Naming Pictures*	.59	.42	.58	.41	.62	.10	.67	.02*	.56	.41	.70	.02*
*Naming Letters*	.68	.10	.72	.03*	.71	.00**	.68	.01*	.69	.01*	.82	.00***
*Word reading*	.81	.00**	.84	.00**	.92	.00***	.89	.00***	.92	.00***	.92	.00***
Phon. Segment. 1	.65	.18	.59	.35	.76	.00***	.66	.03*	.83	.00***	.71	.01*
Phon. Segment. 2	.65	.18	.47	.79	.81	.00***	.75	.00***	.74	.00***	.77	.00*
*Two-Min. Spelling*	.76	.02*	.66	.11	.85	.00***	.72	.00**	.79	.00***	.81	.00***
Backw. Digit Span	.56	.57	.55	.61	.63	.07	.43	.31	.57	.34	.48	.77
*Nons. Pass. Reading*	.79	.00**	.80	.00**	.83	.00***	.89	.00***	.88	.00***	.82	.00***
*Non-Word reading*	.88	.00**	.77	.01**	.91	.00***	.97	.00***	.93	.00***	.98	.00**
*One-Minute Writing*	.76	.02*	.64	.15	.80	.00***	.69	.01*	.76	.00***	.62	.16
Verbal Fluency	.73	.04*	.42	.43	.70	.01*	.38	.10	.61	.12	.53	.74
Semantic Fluency	.54	.69	.40	.30	.65	.04*	.49	.93	.60	.15	.44	.47
PLQ risk score	.84	.00**	.79	.00**	.89	.00***	.89	.00***	.89	.00***	.96	.00**

Italicized DST subtests are part of the PLQ risk score

*p < .05, **p < .01, ***p < .001

In the third grade, only three more subtests had a significant value above .70 for Dutch children: Two-Minutes Spelling, One-Minute Writing, and Verbal Fluency. For the immigrant children, one additional subtest, Naming Letters, had a significant AUC value above .70. In summary, six DST-NL subtests and the PLQ had at least an acceptable and significant AUC score in the Dutch group and four subtests and the PLQ had at least an acceptable and significant AUC value in the immigrant group in the third grade. This finding indicates a rather low agreement between the DST-NL and protocol outcomes.

In the fifth grade, two subtests, Phonological Segmentation 2 and Two-Minutes Spelling, had, in addition to Word Reading, Nonsense Passage Reading, Non-Word Reading, and the PLQ, a significant and acceptable or excellent AUC value for both Dutch and immigrant children. Only for the Dutch children, four other subtests had significant and acceptable or excellent AUC values: Naming Letters (AUC = .71), Phonological Segmentation 1 (AUC = .76), One-Minute Writing (AUC = .80), and Verbal Fluency (AUC = .70). In the fifth grade, nine subtests and the PLQ had at least an acceptable and significant AUC score in the Dutch group, and five subtests and the PLQ had at least an acceptable and significant AUC score in the immigrant group (see Table 4).

In the seventh grade, besides the subtests, Word Reading, Nonsense Passage Reading, Non-Word Reading, and the PLQ, three subtests had significant and acceptable or excellent AUC values in both cultural groups: Phonemic Segmentation 1 (AUC = .83 for Dutch and .71 for immigrant children), Phonemic Segmentation 2 (AUC = .74 for Dutch and .77 for immigrant children), and Two-Minutes Spelling (AUC = .79 for Dutch and .81 for immigrant children). For the Dutch children, one other subtest had a significant acceptable AUC value (One-Minute Writing) and for the immigrant children, two other subtests had a significant acceptable or excellent AUC value (Naming Pictures and Naming Letters; see Table 4). In summary, among seventh graders, seven DST-NL subtests and the PLQ had at least an acceptable and significant AUC score in the Dutch group and eight subtests and the PLQ in the immigrant group. It seems fair to conclude that the DST and protocol outcomes agree more in higher grades and that possible cultural bias is not dealt with in the same way in the two instruments.

As can be seen in Table 4, the number of subtests of the DST-NL with a significant and acceptable or excellent AUC value for both majority group and immigrant children is the highest in the seventh grade (three subtests in the third, five subtests in the fifth, and six subtests in the seventh grade, respectively). More subtests had an acceptable diagnostic performance (AUC score) in the Dutch group in the third and fifth grade, whereas in the seventh grade, more subtests in the immigrant group met this AUC criterion. There were differences in diagnostic performance of the DST-NL in the third, fifth, and seventh grade; the best prediction results were found in the fifth grade for the Dutch children and in the seventh grade for the immigrant children. The prediction value was in most cases higher for the Dutch children, which confirms hypothesis 1 the prediction of dyslexia diagnosis using the DST-NL subtests and therefor the DST-NL risk score is less accurate for immigrant children in the third, fifth, and seventh grade then for Dutch children in the same grades (due to method and item bias). The number of subtests with an acceptable or excellent prediction performance tended to become higher with grade for the immigrant group (four subtests in the third, five in the fifth, and eight in the seventh grade, respectively), as predicted in hypothesis 2 (the convergence between the accuracy of the prediction of the DST-NL subtests scores and therefor the DST-NL risk score and the dyslexia protocol outcomes increase throughout the years of schooling for the immigrant children). The differences in the subtests AUC values between the Dutch and immigrant children became smaller with grade for Word Reading, Phonological Segmentation 2, and Non-Word Reading, because of the increase in prediction performance in the immigrant group and the decrease in the Dutch group. Differences in the subtest AUC values became larger with grade for Naming Pictures, Naming Letters, Phonological Segmentation1, Nonsense Passage Reading, One-Minute Writing, Semantic Fluency, and the PLQ. The AUC values of Two-Minutes Writing, Backward Digit Span, and Verbal Fluency became larger between the third and fifth grade and smaller between the fifth and seventh grade. Some subtests, which are part of the PLQ risk score, were not significant for either immigrant children, or for both groups, or were significant only in a specific grade such as Naming Pictures, Naming Letters, and One-Minute Writing (see Table 4). Verbal subtests, which are not part of the PLQ and without an AUC value that was acceptable across all grades, were Phonemic Segmentation 1 and 2, Backward Digit Span, One-Minute Writing, Verbal Fluency, and Semantic Fluency.

It can be concluded that Naming Pictures, Backward Digit Span, Verbal Fluency, and Semantic Fluency often did not yield similar results for the DST-NL and dyslexia protocol. Naming Letters, Phonemic Segmentation 1 and 2, One-Minute Writing, and Two-Minutes Spelling were somewhat more in agreement with the protocol outcomes, whereas Word Reading, Nonsense Passage Reading, and Non-Word Reading showed most agreement. Although the PLQ discriminated well between children with and without dyslexia diagnosis, we found that several, especially verbal DST-NL subtests, discriminated less for immigrant children, in line with hypothesis 1 and 3 (the verbal subtests of the DST-NL are more difficult for immigrant children in the third, fifth, and seventh grade, even after controlling for the level of Word Lexicon, Parental Education, and grade of first assessment, which suggests cultural bias). The best discriminating subtests involved technical reading aspects, which measure literacy achievement and reading and spelling achievement but probably less the underlying dyslexia source of problems in reading and spelling achievement such as phonological awareness and rapid naming.

## Multilevel modeling

Word Lexicon can be interpreted as a proxy for knowledge of the Dutch language and culture, which could be an important confounding variable to understand score differences between the mainstream and immigrant children and potential bias threats in the assessment of dyslexia, using the DST-NL. To address the role of Word Lexicon in a more detailed manner, a two-level Hierarchical Linear Multilevel (HLM) modeling was used. This analysis (including the repeated measures) addressed individual growth, in subtest scores with grade (as independent variable, level 1) as a function of the following (level 2) predictors: Cultural Background (Dutch or immigrant), Dyslexia Diagnosis (yes or no), assessed following Blomert’s (2006) and the NRD’s Dutch Dyslexia Protocol (, 2013), Parental Education Level, and grade first DST-NL assessment. In the second analysis, Word Lexicon was added as predictor. The results are presented in Table 5; mean scores per subtest are presented in Table 6. We were particularly interested in shifts in regression coefficients and their significance after introducing Word Lexicon. The latter variable was significant for each dependent variable and invariably in the expected direction. It is remarkable that introducing Word Lexicon as a predictor had no noticeable influence on the pattern of significance of the dyslexia diagnosis, parental education, and first grade of assessment, but had a major impact on cultural background. Naming Letters and Semantic Fluency lost their significance after introducing Word Lexicon, but the opposite pattern was more common; Phonological Segmentation 2, Two-Minutes Spelling, Backward Digit Span, Nonsense Passage Reading, One-Minute Writing, and Verbal Fluency became significant. The analysis suggests that if the influence of lexical knowledge is “taken away” by introducing Word Lexicon, two semantic subtests lose their significance, Naming Letters and Semantic Fluency, but most subtests start to become more strongly associated (namely Phonological Segmentation 2, Two-Minutes Spelling, Backward Digit Span, Nonsense Passage Reading, One-Minute Writing, and Verbal Fluency). Cultural and semantic knowledge had an important association with these subtests, in this research, which makes these subtests less suitable for assessment in our multicultural group as lexical knowledge seems to confound their scores.

**Table 5:** Regression coefficients from the HLM analysis of DST scores and all predictors without and with Word Lexicon

	Cultural background		Dyslexia diagnosis		Parental education		Grade first assessment		Word Lexicon
	Without	With	Without	With	Without	With	Without	With	
Naming Pictures	1.77	− .33	2.20	1.79	*.83*	1.57*	− .99*	*− .57*	− .10***
Naming Letters	3.39*	*.48*	5.73***	5.03**	− .98	.14	− 1.42**	− 1.14**	− .16***
One-Minute Reading	− .91	− 7.08	41.47***	42.08***	− 2.54	− .04	− 2.10*	− 2.43*	− .37***
Phonological Segmentation 1	− .19	.16	− 1.25***	− 1.18***	− .01	− .15	.29***	.24***	.02***
Phonological Segmentation 2	*− .39*	.59*	− .99**	− 1.20**	*− .01*	− .33*	2.19***	1.46***	.05***
Two-Minutes Spelling	*− .14*	2.29***	− 2.01**	− 2.23**	*− .01*	− .96**	4.44***	3.09***	.11***
Backward Digit Span	*.01*	.35*	− .10	− .07	.12	.02	.26***	.12*	.02***
Nonsense Passage Reading	*1.65*	4.06***	− 9.24***	− 8.63***	.11	− .67	1.23***	1.09**	.11***
Non-Word Reading	*− 16.28*	− 39.64***	128.32***	119.03***	*3.46*	11.29*	− 17.41***	− 9.57**	− 1.19***
One-Minute Writing	*− .01*	1.57**	− 2.21**	− 2.02**	*− .07*	− .57*	3.01***	2.08***	.07***
Verbal Fluency	*.07*	1.29**	− .39	− .21	.22	− .11	1.07***	.56***	.06***
Semantic Fluency	− 1.86***	*− .64*	− .70	− .41	.15	− .25	.99***	.53**	.06***
PLQ	− 12.75	− 47.33	171.74***	168.93***	− 4.50	10.26	− 5.55	− 8.04	− 1.88***

Cultural background means Dutch (scored as 1) or immigrant (scored as 2). Italicized numbers refer to predictors that differ in significance when lexicon is (nor) included

*p < .05, **p < .01, ***p < .001

**Table 6:** Raw mean scores of Dutch and immigrant non-dyslexic and dyslexic third, fifth, and seventh graders

Tests	3rd grade				5th grade				7th grade			
	Dutch		Immigrant		Dutch		Immigrant		Dutch		Immigrant	
	Non-dyslexic	Dyslexic	Non-dyslexic	Dyslexic	Non-dyslexic	Dyslexic	Non-dyslexic	Dyslexic	Non-dyslexic	Dyslexic	Non-dyslexic	Dyslexic
Naming Pictures	*60.00*	*62.89*	*65.19*	*70.20*	*48.01*	*51.10*	*48.93*	*54.11*	*43.70*	*45.42*	*43.43*	*47.93*
Naming Letters	*51.68*	*71.56*	*56.41*	*69.40*	*32.95*	*45.14*	*41.66*	*49.21*	*25.03*	*28.83*	*27.87*	*36.29*
One-Min. Reading	*197.35*	*312.22*	*212.40*	*310.90*	*67.30*	*147.38*	*76.52*	*133.16*	*46.20*	*90.92*	*49.62*	*92.29*
Phon. Segm. 1	6.62	5.22	5.93	4.80	10.37	8.24	9.81	8.42	11.00	9.08	10.52	9.57
Phon. Segm. 2	0.76	0.00	0.31	0.00	7.09	2.67	5.25	2.42	8.41	5.79	7.69	4.50
Two-Min. Spelling	4.65	2.67	3.97	2.90	16.30	10.33	15.76	12.26	24.27	20.29	24.20	19.93
Backw. Digit Span	3.35	3.22	3.08	2.80	4.36	3.86	4.27	4.58	4.95	4.71	4.76	4.86
Nons. Pass. Reading	32.88	19.44	32.49	20.80	58.34	47.05	58.73	48.53	64.28	54.33	66.36	56.00
Non-Word Reading	*272.56*	*414.22*	*285.55*	*385.30*	*220.32*	*389.05*	*197.75*	*377.84*	*152.55*	*294.75*	*141.71*	*266.79*
One-Min. Writing	6.32	4.11	5.65	4.50	14.11	9.38	14.34	11.11	17.95	13.79	17.58	15.57
Verbal Fluency	6.03	3.44	5.53	6.40	10.01	7.14	8.70	10.00	12.15	10.71	11.75	11.43
Semantic Fluency	11.97	11.33	9.80	10.90	16.32	14.24	13.74	13.84	18.53	17.25	15.85	16.79
PLQ risk score	69.00	53.56	67.68	54.80	72.51	49.29	69.45	49.89	72.31	52.46	72.46	50.43

The four italicized subtests are speed measures where lower scores point to better performance and lower dyslexia risk

In summary, in both analyses (ROC and HLM), we concluded that having a dyslexia diagnosis according to the dyslexia protocol is well predicted by several subtests and the PLQ, with and without controlling for Word Lexicon: Naming Letters, One-Minute Reading, Phonological Segmentation 1 and 2, Two-Minutes Spelling, Nonsense Passage Reading, Non-Word Reading, and One-Minute Writing. These subtests were not or less associated with Cultural Background or Word Lexicon achievement in the HLM analysis. More specifically, Phonological Segmentation 1 and 2 had an acceptable predictive ability in the fifth and seventh grade in the ROC analyses but not in the third grade. These subtests were less effective in the third grade. The subtests, Naming Pictures, Backward Digit Span, Verbal Fluency, and Semantic Fluency, could not be predicted by a diagnosis of dyslexia irrespective of grade, Cultural Background, or Word Lexicon achievement.

## Discussion

The purpose of this research was to examine to what extent it is possible to detect Dutch and immigrant children at risk for dyslexia with the same instrument (DST-NL); we set out to make a reasonable case for the decrease of cultural bias in the DST-NL for immigrant children across the third, fifth, and seventh grades, taking into account the differences in stage of reading development per grade. Assessing dyslexia in a group of immigrants creates a serious problem due to presence of cultural bias and the confounding of language knowledge and dyslexia problems. There is no easy way to resolve this conundrum. Still, despite these problems, we suggest various ways forward.

Different cultural and language factors can have an influence on test scores; screening for dyslexia could be more difficult in immigrant children because of these differences (Gollan & Brown, 2006; Gollan et al., 2002; Verpalen & van de Vijver, 2011, 2015). We found associations between the protocol outcome and some DST-NL subtest scores (hypothesis 1: the prediction of dyslexia diagnosis using the DST-NL subtests and therefor the DST-NL risk score is less accurate for immigrant children in the third, fifth, and seventh grade than for Dutch children in the same grades). The DST-NL was less useful in the third grade for our Dutch and immigrant participants, which made it difficult to detect dyslexia early in arguably the most important period in the reading development of the child using the DST-NL, which limits the opportunity for early intervention. Subtest scores were most accurate in the fifth grade and least accurate in the third grade, contrary to our expectation (hypothesis 2: the convergence between the accuracy of the prediction of the DST-NL subtest scores and therefore the DST-NL risk score and the dyslexia protocol outcomes increase throughout the years of schooling for the immigrant children). Probably, dyslexia is easier to identify in the fifth or higher grade of our population, because differences between differences in reading skills between dyslexic and non-dyslexic children become more pronounced with age and grade. Various verbal subtests (Naming Pictures, Backward Digit Span, and Verbal and Semantic Fluency) do not seem to be useful to detect a dyslexia risk in Dutch and even more so in immigrant children (hypothesis 3: the verbal subtests of the DST-NL are more difficult for immigrant children in the third, fifth, and seventh grade, even after controlling for the level of Word Lexicon, Parental Education, and the grade of first assessment).

Several subtests of the DST-NL showed score differences between Dutch and immigrant children subtest scores present in our population that challenged the cross-cultural suitability of the PLQ. The subtests of the PLQ are more based on technical aspects of reading and spelling (achievement in reading and spelling) and less on the underlying cause of problems in reading and spelling (rapid naming is included but phonological awareness is no part of the risk score). The DST-NL seems useful to detect literacy problems (which are always present in dyslexic children), but may be less successful in detecting the dyslexia risk as underlying cause in our multicultural population. Probably, the composition of the DST-NL with the technical literacy tasks within the PLQ and the phonological tasks without the PLQ is not obvious because of the important role of phonological awareness in detecting dyslexia. The Dutch protocol (NRD, 2013) includes cut-off criteria for diagnosing dyslexia in three domains: phonological awareness tasks, the grapheme-phoneme association tasks, and rapid naming tasks. The DST-NL risk indicator (PLQ) has only two naming tasks relevant for these criteria included (Naming Pictures and Naming letters), from which only Naming Letters seemed to converge more with protocol outcomes.

The level of Word Lexicon, as a proxy for knowledge of the Dutch language and culture, had an association with all subtests and the PLQ, whereas the level of Parental Education was associated with only a few subtests. Differences in scores could be explained because of these associations, the immigrant group in our research had significantly lower scores on Word Lexicon and Parental Education, compared to the Dutch group in this research, in all grades (3rd, 5th, and 7th). Although researchers have shown that the experience of speaking two languages (with two lexical systems) may have positive implications for cognitive ability, enhancing executive-control functions across the lifespan, negative consequences of bilingualism have also been found specifically for verbal knowledge and some specific skills, such as smaller vocabularies and less-rapid access to lexical items (Bialystok & Craik, 2010; Michael & Gollan, 2005). These researchers found bilinguals to be slower, to commit more errors in picture naming (even in their dominant language), to obtain lower scores on verbal fluency tasks and to demonstrate more interference in lexical decisions over the life span (Bialystok & Craik, 2010; Michael & Gollan, 2005). These negative results could be a consequence of the process of inhibition: the bilingual child does not only have to perform the task (e.g., to name the picture) but also has to select a language in which to name the picture and to repress the other language (Green, 1998). Another possible explanation could be that the links in the lexical system between concepts and lexical representations specific to each language are weaker as a consequence of using two languages in everyday life. Bilinguals have to learn and use twice as many items as monolinguals, and they use these words less often than monolinguals, thus the connection within the lexical system between concepts and phonological representations are weaker (Gollan & Acenas, 2004; Gollan et al., 2002). Evidence of these bilingual “processing costs” comes from studies on response time during picture naming and verbal fluency tasks, such as slower naming of pictures and reduced category and letter fluency (Gollan et al., 2002; Gollan & Silverberg, 2001). In line with these findings, we observed differences in the verbal fluency, semantic and naming tasks of the DST-NL, and we also found an association between Word Lexicon and these subtests. We found associations between some subtests and Cultural Background. Word Lexicon was associated with Cultural Background and having a dyslexia diagnosis. The influence of Word Lexicon and cultural knowledge in testing immigrant children with the DST-NL is confirmed in these findings. Our study shows a limited applicability of the DST-NL for the assessment of dyslexia risk of our population, comprising children who do not speak Dutch as their mother tongue and are not very familiar with the Dutch culture. Literacy problems are detected well with the DST-NL in this population. The tests could probably be more accurate in predicting dyslexia risk if the verbal tasks rely less on knowledge of the Dutch language or culture (for example, names of Dutch persons or pictures could be substituted by Dutch high frequency words).

A limitation of our research is the small number of immigrant children with a high level of Word Lexicon. It is another limitation of our study that we used a clinical diagnosis as the criterion to decide whether a child was dyslexic. This clinical judgment may also be susceptible to cultural bias. It is reasonable to expect that if there would be any bias in this judgment, it would go in the same direction as found in the DST-NL subtest scores. As a consequence, our estimate of cultural bias may be conservative. A criterion measure with demonstrated adequacy in a multicultural population would be a better reference point.

Researchers of different countries have highlighted the critical need for a culture-informed way of dyslexia assessment (Elbeheri & Everatt, 2007; Peer & Reid, 2016). There is still a lack of diagnostic tests to distinguish between differences in reading and spelling achievement related to bilingualism, cultural differences, and language impairments such as dyslexia (De Abreu, Baldassi, Puglisi, & Befi-Lopes, 2013; Goulandis, 2003). Our research showed the same issues and made clear that to identify dyslexia in bilingual children, it is important to take into account the relevant features of their home language which can explain some mistakes in answers. Test scores have to be interpreted very carefully. Van der Leij (2004) refers to the importance of the basic mapping process of phonemes and graphemes, which is important in the learning process of reading and spelling in all languages. To develop an instrument or methods to identify dyslexia, it is necessary to determine this process, which has a similar neurocognitive basis across languages (Paulesu et al., 2001), and to realize that the way dyslexia manifests seems to vary across languages depending on the specific language features (e.g., the level of transparence). So, even when dyslexia is a universal phenomenon, its manifestations will be influenced by specific language features, which implies that culturally shared underlying psychological constructs involved in dyslexia, such as phoneme-grapheme mapping, should be assessed with regard to their specific manifestation in each language. The learning effect of training the phonological awareness tasks and phoneme/grapheme mapping tasks in the second language provides additional information in the process of identifying dyslexic children. Treatment seems to be more effective in non-dyslexic children and less in dyslexic children. Researchers of the Dyslexia and Multilingualism Project (Montimore et al., 2012) studied the effect of treatment in dyslexic and non-dyslexic children and advised to treat all children with reading and spelling problems and classify them as children with special needs, which makes a diagnosis less necessary. These alternative ways of view could help, in every country, children with reading and spelling problems regardless of having a diagnosis or cultural background.

On the other hand, researchers argued that it has to be possible to assess dyslexia in young children, even when they at preschool age (e.g. Van der Leij, 2013; Elbro & Petersen, 2004; Regtvoort & Van der Leij, 2007; Van Otterloo et al., 2009) as similar phonological and rapid naming tasks are useful to predict dyslexia in first and second language learners when they have been exposed to literacy instruction (Geva, 2000; Geva & Clifton, 1993; Geva & Siegel, 2000; Gholamain & Geva, 1999). This means that it could be possible that adaptations to the studied phonological and naming tasks, following the guidelines of the International Test Commission (ITC, 2005), could make the tasks useful for young children, regardless of their linguistic background. In these test, adaptation items are adjusted if and when needed. The need to adapt items comes from a conceptual analysis of the adequacy of the items to tap into dyslexia-relevant processes, combined with a statistical evaluation of the adequacy of the test evaluations. Clearly, more research is needed to indicate whether it is possible to create tasks useful to assess dyslexia in a young multicultural population that could contribute to an accompanying protocol.
